# Risk of Multiple Myeloma in Rheumatoid Arthritis: A Meta-Analysis of Case-Control and Cohort Studies

**DOI:** 10.1371/journal.pone.0091461

**Published:** 2014-03-13

**Authors:** Kaini Shen, Gufeng Xu, Qing Wu, Daobin Zhou, Jian Li

**Affiliations:** 1 Department of Hematology, Peking Union Medical College Hospital, Chinese Academy of Medical Sciences and Peking Union Medical College, Beijing, People’s Republic of China; 2 Zhejiang University, School of Medicine, Hangzhou, Zhejiang, People’s Republic of China; 3 Division of Health Sciences Research, Mayo Clinic, Scottsdale, Arizona, United States of America; IRCCS National Cancer Institute, Italy

## Abstract

**Objectives:**

multiple myeloma is a malignant neoplasm of plasma cells mainly affecting elderly patients. Despite the wealth of information available on therapeutic strategies, the etiology and pathogenesis of myeloma remain unclear. In the current study, a meta-analysis was conducted to assess the possible association between rheumatoid arthritis and myeloma.

**Methods:**

a literature search was conducted with PubMed, EMBASE and Web of Science for relevant studies published by December 25, 2013. Additionally, we searched annual meeting abstracts of the American Society of Hematology from 2004 to 2013. Only original studies that investigated the association between rheumatoid arthritis and myeloma were included. In total, 8 case-control and 10 cohort studies were identified for analysis.

**Results:**

the meta-estimate of the association between rheumatoid arthritis and myeloma was 1.14 (95% CI, 0.97–1.33) overall, with significant heterogeneity among studies. The relationship between myeloma and other autoimmune diseases was additionally examined from available data. Our results showed that myeloma risk is increased 1.31 to 1.65-fold in pernicious anemia and 1.36 to 2.30-fold in ankylosing spondylitis patients.

**Conclusion:**

Rheumatoid arthritis does not appear to alter the risk of myeloma, while between-study heterogeneity analyses suggest caution in the interpretation of results. Pernicious anemia and ankylosing spondylitis may be potential risk factors for myeloma development. Future large-scale epidemiological studies with reliable exposure biomarkers are necessary to establish the possible contribution of autoimmune disorders to multiple myeloma.

## Introduction

Multiple myeloma (MM) is one of the most common hematologic malignancies with poor prognosis, characterized by neoplastic proliferation of monoclonal plasma cells. The incidence of MM in developed countries is about 5–7 cases per 100 000 individuals [1]. Despite extensive research efforts over the last two decades, the risk factors of MM remain to be established. Numerous case-control and cohort studies to date have suggested that several environmental factors may be associated with the risk of MM, such as ionizing radiation [2], benzene [3] and agricultural occupation [4]. The relationship between autoimmune disease and hematopoietic malignancies was initially postulated in 1964 [5]. However, the issue of whether autoimmune diseases increase the incidence of MM remains controversial.

On the basis of animal models of MM, it is suggested that repeated antigenic stimulation of the immune system plays an important role in MM development [6], [7]. A number of studies have confirmed an association of lymphoma with autoimmune conditions [8]–[12], including rheumatoid arthritis (RA), systemic lupus erythematosus (SLE), and autoimmune hemolytic anemia, although the underlying pathogenesis is unclear. Similarly, the relationship between MM and autoimmune disease has been a long-term subject of investigation, but inconsistent results have been obtained to date.

The main aim of this meta-analysis was to estimate the comparative risk of developing MM in RA patients versus the general population. Two additional goals were (1) to explore the variation in the association of RA and MM among subgroups of interest and (2) to investigate if there is an association between MM and other autoimmune diseases.

## Methods

### Search Strategy

A literature search was conducted with PubMed (from January 1, 1951), EMBASE (from January 1, 1955) and Web of Science (from January 1, 1970) using the following search terms: rheumatoid arthritis, autoimmune disease, etiology, epidemiology, risk factors, multiple myeloma, cancer, malignancy. The final search strategy in PubMed was attached as appendix. The last literature search was performed on December 25, 2013. We also searched annual meeting abstracts of the American Society of Hematology from 2004 to 2013 to identify eligible unpublished data. All references cited in the retrieved articles were additionally reviewed to indentify additional eligible studies. Inclusion criteria were as follows: human participants without limitation of sex or geographic location, case-control or cohort studies, prior medical history of RA as exposure, MM as outcome, studies that reported relative risk (RR), standardized incidence ratio (SIR) or odds ratio (OR) of MM patients with prior history of RA, publication in English. In addition, the selection of cohort studies for inclusion was made regardless of specific RA management strategies. When duplicate reports for the same population and data source were eligible, we chose the original reports with the largest sample size. Case series, case reports, in vitro and animal studies were excluded. Eligibility assessment was performed independently by two reviewers, and disagreements resolved by consensus.

### Data Extraction

Two authors performed data extraction independently, and any discrepancies were addressed by discussion and re-evaluation. We obtained information on the author, year of publication, country of origin, source of case, control and cohort, controlled factors, diagnosis criteria and treatment regimen for RA. Cohort size, number of cases, cohort duration, SIR and 95% confidence intervals (CIs) or sufficient data to allow calculation of these numbers were additionally necessary for cohort studies. For case-control studies, the exact numbers of cases and controls by RA, OR and 95% CIs were required. For the purpose of ascertaining the relationship between MM and other autoimmune diseases, related data were recorded, where available, according to the above extraction principles.

The Newcastle-Ottawa Scale (NOS), developed for evaluating the quality of nonrandomized studies [13], was used by two independent reviewers to assess the methodological quality of each study, and the scores subsequently used in subgroup analysis. A score of ≤5 was considered as relative low quality [14].

### Statistical Analyses

In case-control studies, OR and 95% CIs for MM risk factors were directly extracted from original research papers or calculated when not provided. The primary outcome for cohort studies was SIR and corresponding 95% CI. In cases where SIRs were not specifically reported, calculations were made from the number of observed MM divided by the number of expected cases in the general population provided by authors, and 95% CI determined using the standard error of the natural logarithm of SIR, estimated from the inverse of the square root of the observed number of cases. The measure of interest was RR, estimated from ORs in case-control studies and SIRs in cohort studies. Since the incidence of MM is low, SIR and OR produce similar estimate of RR, thus we present all results as RR for simplicity [15]–[17]. Between-study heterogeneity was examined using a chi-square test of heterogeneity and *I* ^2^ measure of inconsistency. P-values less than 0.1 or the *I* ^2^ statistic greater than 50% were considered statistically significant [18]. Under these conditions, data were pooled based on the method of Dersimonian and Laird under a random effects model otherwise under fixed effects model. Two-tailed p≤0.05 was considered statistically significant for all analyses.

To evaluate publication bias, we constructed a funnel plot and applied Begg’s test. Trim and Fill analysis was used to estimate the number of missing studies and their potential effects on outcomes. Sensitivity analyses were additionally conducted to ascertain the robustness of our findings. The influence of RA was examined by excluding studies restricted to elderly patients (≥65 years) or single-sex participants and those that failed to control important confounders. All meta-analyses were conducted with Stata 12.0.

## Results

**Figure 1:**
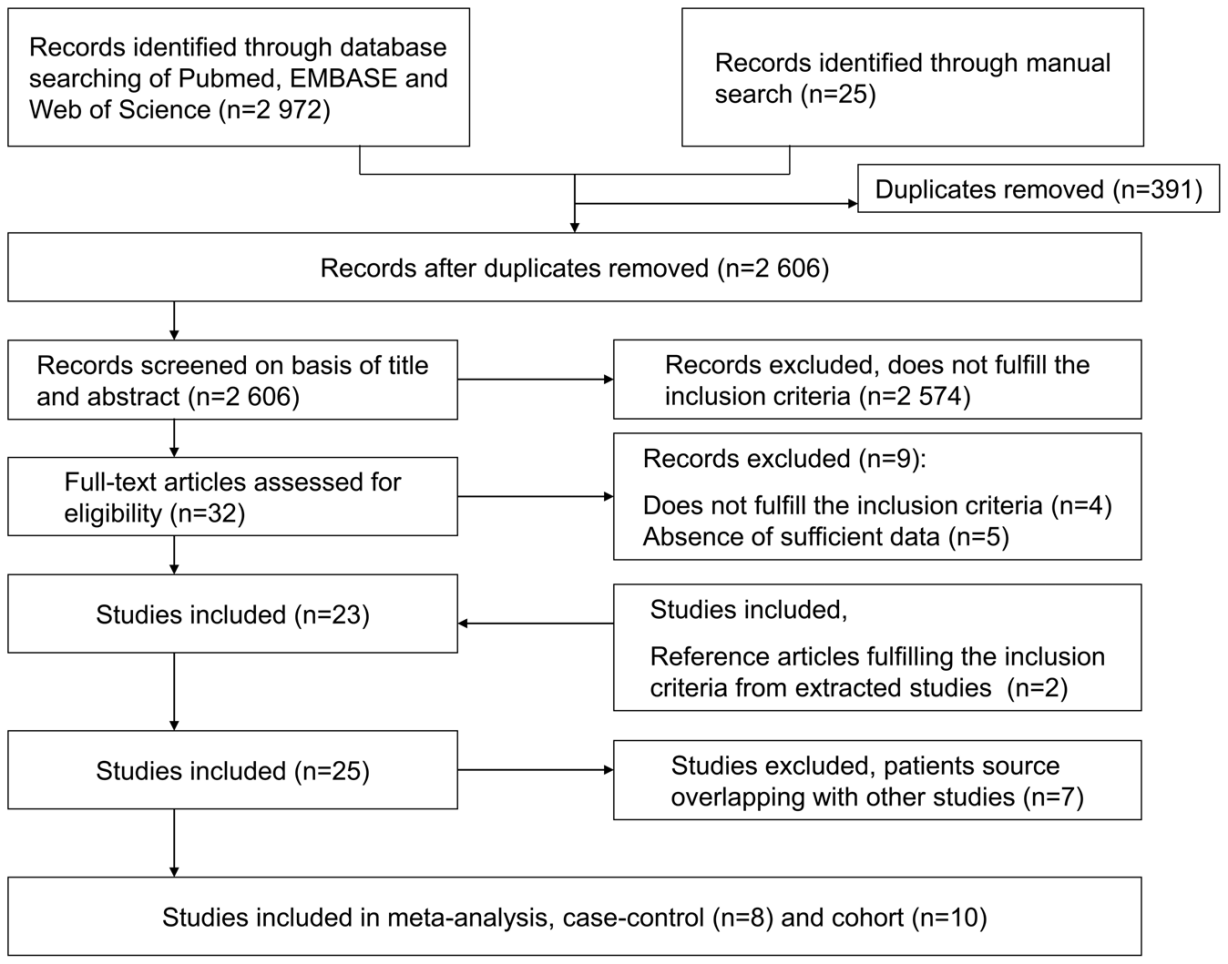
Flow diagram depicting the identification and selection of eligible case-control and cohort studies.

Abstract evaluation yielded thirty-two potential studies for analysis (Figure 1), of which five [19]–[23] were excluded, since they failed to provide sufficient data for calculation. Twenty-three case-control and cohort studies were included after full text assessment. Another two publications [24], [25] were incorporated after examining references from the extracted articles. Among the 25 studies, seven [12], [26]–[31] were subsequently excluded for overlap in case or cohort resources. Consequently, our meta-analysis consisted of eight [24], [25], [32]–[37] case-control and ten [10], [38]–[46] cohort studies.

All the studies published from 1982 to 2013 were performed in USA or Europe, except one case- control study from New Zealand [25]. There was slight overlap in the patient populations of two studies [38], [40] from Finland. Landgren et al. [36] limited the study subjects to women, while the groups of Pearce [25] and Brown [45] mainly focused on the role of prior autoimmune disorders in male MM. The NOS scores varied from 5 to 7 in all case-control studies, with the exception of the reported score of 3 by Landgren and co-workers [36] as a result of unsatisfactory comparability. Since almost all the cohort studies used the expected cancer incidence calculated from national cancer rates as a comparison instead of the non-exposed cohort, NOS scores were relatively low, ranging from 4 to 5.

In the eight case-control studies, OR was all obtained from the published articles or calculated from the numbers of cases and controls by RA. In four cohort studies [10], [38], [43], [44], the SIRs and 95% CIs were estimated from the number of observed MM and expected MM incidence provided by authors, while others were directly extracted from the original article. Only three studies [41], [42], [44] reported the type of treatment for RA before MM diagnosis. All important information is presented in Tables 1 and 2.

**Table 1:** Characteristics of case-control studies enrolled in the meta-analysis.

Author	Year	Country	Sex	Controls	Cases		Controls		OR (95% CI)	Controlled Factors
					RA	Non-RA	RA	Non-RA		
Pearce NE	1986	New Zealand	M	Population	4	72	7	308	2.30 (0.60–8.00)	Age, registration year
Cohen HJ	1987	USA	M+F	Hospital	62	91	203	210	0.70 (0.48–1.03)	Age, gender, race, disease status
Boffetta P	1989	USA	M+F	Population	39	89	164	348	0.90 (0.60–1.50)	Age, gender, race, residence
Lewis DR	1994	USA	M+F	Population	64	509	241	1 890	0.80 (0.60–1.10)	Age, gender, race
Vlajinac HD	2003	Yugoslavia	M+F	Hospital	12	85	5	95	2.60 (0.90–7.60)	Age, gender, residence
Landgren O	2006	USA	F	Population	7	172	9	682	2.30 (0.80–6.50)	none
Anderson LA	2009	USA	M+F	Population	263	9 211	3 289	119 242	1.00 (0.90–1.10)	Gender, year of diagnosis, age at diagnosis
Lindqvist EK	2011	Sweden	M+F	Population	111	19 001	567	74 841	0.80 (0.60–0.90)	Age, gender, residence

Abbreviations: RA, rheumatoid arthritis; OR, odds ratio; CI, confidence intervals; M, male; F, female.

**Table 2:** Characteristics of cohort studies enrolled in the meta-analysis.

Author	Year	Country	Sex	Cohort Size	Number of MM	SIR (95% CI)	Follow-up Duration (PY)
Isomaki HA	1982	Finland	M+F	46 101	28	2.20 (1.52–3.19)	213 911
Mellemkjaer L	1996	Denmark	M+F	20 699	21	1.10 (0.70–1.70)	144 421
Kauppi M	1997	Finland	M+F	9 469	8	1.20 (0.50–2.30)	65 391
Thomas E	2000	England	M+F	26 623	38	1.66 (1.21–2.28)	151 987
Setoguchi S	2006	USA	M+F	7 830	19	2.00 (1.26–3.12)	33 410
Herrinton LJ	2008	USA	M+F	2 982	2	2.36 (0.28–8.50)	7 791
Brown LM	2008	USA	M	–	94	1.17 (0.94–1.45)	27 years
Parikh-Patel A	2009	USA	M+F	84 475	64	0.90 (0.70–1.15)	405 540
Hemminki K	2012	Sweden	M+F	72 309	81	0.88 (0.70–1.09)	731 954
Dreyer L	2013	Denmark	M+F	7 159	4	1.76 (0.66–4.69)	24 811

Abbreviations: M, male; F, female; MM, multiple myeloma; SIR, standardized incidence ratio; CI, confidence intervals; PY, person years.

–: Not mentioned.

**Figure 2:**
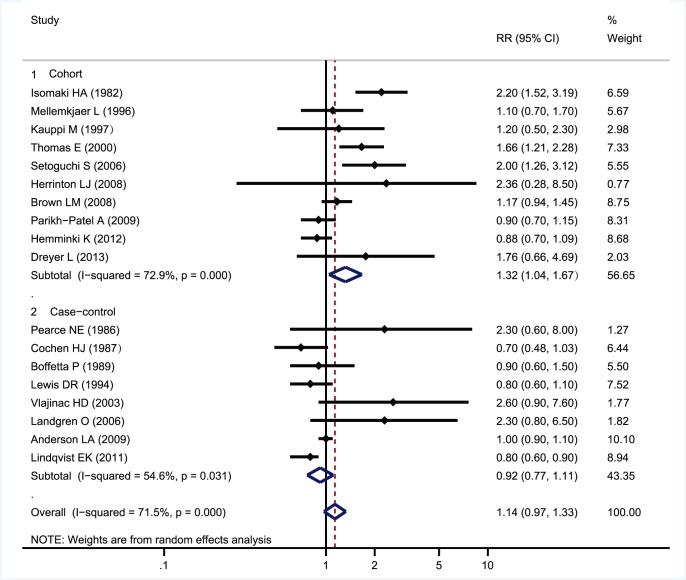
Forest plot of studies on multiple myeloma development risk in rheumatoid arthritis.

The RRs of MM for RA versus non-RA patients overall and by study design was presented (Figure 2). The random-effects pooled RRs for MM were 1.32 (95% CI, 1.04–1.67) for cohort studies and 0.92 (95% CI, 0.77–1.11) for case-control studies. Compared with non-RA patients, RA patients showed an RR of 1.14 (95% CI, 0.97–1.33) regardless of study type, possibly suggesting lack of association between RA and MM. However, overall analysis revealed significant between-study heterogeneity (p<0.1; *I* ^2^ = 71.5%).

Sensitivity analysis was presented in Table 3. After excluding studies restricted to elderly patients (≥65 years) or single-sex participants and those that failed to adjust important factors, overall RR decreased slightly to 1.09, 1.11 and 1.11, respectively.

**Table 3:** Sensitivity analysis of the association between multiple myeloma and rheumatoid arthritis according to different exclusion criteria.

Studies included	Studies (N)	RR (95% CI)	p value	*I* ^2^ (%)
All studies	18	1.14 (0.97–1.33)	<0.001	71.5
Studies without age restriction of participants^a^	17	1.09 (0.94–1.27)	<0.001	68.7
Studies with both male and female participants^b^	15	1.11 (0.94–1.32)	<0.001	74.1
Studies that controlled for important confounding factors^c^	16	1.11 (0.95–1.30)	<0.001	73.1

a Excludes the study by the group of Setoguchi [41].

b Excludes studies by the groups of Pearce [25], Landgren [36] and Brown [45].

c Excludes studies by the groups of Pearce [25] and Landgren [36].

Abbreviations: N, number; RR, relative risk; NS, not significant; CI, confidence interval.

To investigate the source of heterogeneity, subset analysis by study design, publication year, study quality, geographic region, control type and average follow-up duration was conducted. Statistical association between RA and MM was only found to be significant in studies with relatively low quality (RR = 1.29, 95% CI, 1.02–1.64) and cohort studies with relatively low quality (RR = 1.36, 95% CI, 1.02–1.81). Overall, in studies with relatively higher quality according to NOS, as well as those published after 2000, RA was not associated with increase in MM risk (RR = 0.95, 95% CI, 0.79–1.14 and RR = 1.15, 95% CI 0.96–1.37, respectively). Notably, subgroup analyses failed to define the reason for heterogeneity (Table 4).

**Table 4:** Meta-analysis of multiple myeloma incidence by subgroup.

	Subgroup	Analyses (N)	RR (95% CI)	p-value	*I* ^2^ (%)
Overall	Study design	Cohort (10)	1.32 (1.04–1.67)	<0.001	72.9
		Case-control (8)	0.92 (0.77–1.11)	0.031	54.6
	Publication year	Before 2000 (7)	1.10 (0.77–1.59)	<0.001	75.7
		After 2000 (11)	1.15 (0.96–1.37)	<0.001	71.3
	Study quality	NOS≤5 (13)	1.29 (1.02–1.64)	<0.001	72.7
		NOS>5 (5)	0.95 (0.79–1.14)	0.024	64.3
	Region^a^	USA (9)	1.02 (0.86–1.22)	0.008	61.4
		Europe (8)	1.30 (0.93–1.79)	<0.001	80.9
Cohort	Publication year	Before 2000 (3)	1.48 (0.90–2.45)	0.047	67.3
		After 2000 (7)	1.25 (0.96–1.61)	0.002	72.0
	Study quality	NOS≤5 (9)	1.36 (1.02–1.81)	<0.001	75.9
		NOS>5 (1)	1.17 (0.94–1.45)	–	–
	Region	USA (4)	1.25 (0.89–1.77)	0.017	70.4
		Europe (6)	1.37 (0.95–1.97)	<0.001	77.7
	Mean follow-up^b^	<5 years (5)	1.63 (0.97–2.74)	<0.001	80.4
		≥5 years (4)	1.17 (0.82–1.66)	0.015	71.2
Case-control	Publication year	Before 2000 (4)	0.81 (0.65–1.02)	0.352	8.1
		After 2000 (4)	1.02 (0.77–1.36)	0.021	69.1
	Study quality	NOS≤5 (4)	1.14 (0.71–1.83)	0.060	59.5
		NOS>5 (4)	0.88 (0.71–1.10)	0.046	62.5
	Region^a^	USA (5)	0.91 (0.74–1.11)	0.116	45.9
		Europe (2)	1.28 (0.41–3.96)	0.033	77.9
	Control type	Population (6)	0.92 (0.78–1.10)	0.088	47.8
		Hospital (2)	1.22 (0.34–4.36)	0.023	80.6

Abbreviations: N, number; M, male; F, female; RR, relative risk; NOS, Newcastle-Ottawa Scale.

a One case-control study conducted in New Zealand was excluded from analysis.

b One study with unknown mean follow-up duration was excluded from analysis.

–: Cannot be calculated.

**Figure 3:**
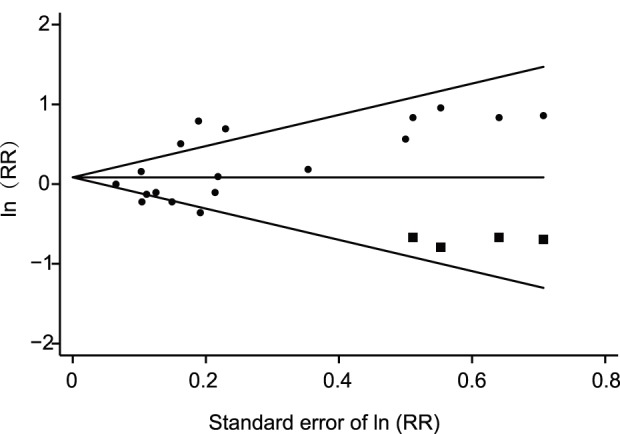
Funnel plot for case-control and cohort studies (dots: original data; squares: filled data).

Publication bias in this meta-analysis was suspected as indicated from the funnel plot and Begg’s test (p = 0.081). However, RR values remained similar after Trim and Fill correction for possible missing data (RR = 1.14, 95% CI 0.97–1.33 before Trim and Fill versus RR = 1.09, 95% CI 0.93–1.27 after Trim and Fill) (Figure 3).

**Figure 4:**
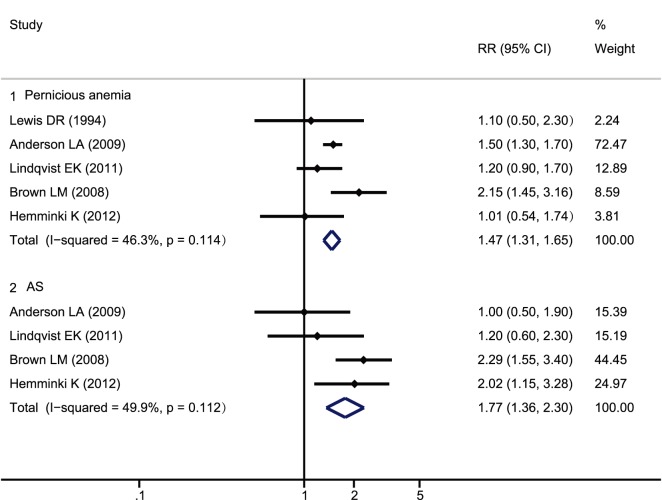
Forest plot of studies on multiple myeloma development risk in pernicious anemia and AS.

The reported relevance of other autoimmune diseases, including pernicious anemia and ankylosing spondylitis (AS), in MM incidence is presented (Figure 4). Patients with pernicious anemia and AS displayed a 1.31- to 1.65-fold and 1.36- to 2.30-fold increase in MM risk, respectively. In contrast, MM development appeared to be unrelated to prior medical history of psoriasis (RR = 0.91, 95% CI, 0.78*–*1.05), SLE (RR = 1.18, 95% CI, 0.88–1.60), Sjögren syndrome (SS) (RR = 1.22, 95% CI 0.83–1.78) or polymyositis/dermatomyositis (RR = 1.37, 95% CI 0.86–2.17). The between-study heterogeneity analyses were all not statistically significant.

## Discussion

Our meta-analysis results suggest that MM seems not more common in patients with a past history of RA, consistent with our sensitivity data. However, subgroup analysis revealed that RA patients are more likely to suffer from subsequent MM in cohort studies (RR = 1.32, 95% CI 1.04–1.67). Information on prior autoimmune diseases in case-control studies was mostly obtained from self-reports through interviews or questionnaires, while in cohort studies, investigators relied mainly on linkage records, which probably attributed to the differences between the two types of studies. Notably, subset analysis confirmed that statistical association between RA and MM was only significant in studies with NOS score≤5, further indicating that RA might not act as risk factor for MM.

A number of studies have explored whether repeated or chronic antigenic stimulation leads to hematopoietic malignancies. Some researchers [31] reported elevated risk for MM in patients with RA, while others [47], [48] reached different conclusions. An antigenic stimulation hypothesis was proposed to explain the excessive risk of MM in patients with autoimmune diseases. Chronic immune stimulation resulting from autoimmune disorders, with its associated lymphocyte activation, tends to induce uncontrolled proliferation of malignant plasma cells, leading to MM [49]. Since no biologic marker has been developed that can effectively measure lifetime immune stimulation, this theory remains controversial. Meanwhile, evidence from previous studies indicates that increased risk could be a consequence of therapy, rather than the disease itself. For instance, higher risk of non-Hodgkin’s lymphoma and MM has been reported following use of steroids [36], [50], which are important for autoimmune disease treatment. Similarly, cytotoxic and immunosuppressive drugs may suppress immunodefense against malignant cells and lead to oncogenesis [51]. Moreover, there may be a common genetic or environmental susceptibility in autoimmune diseases and plasma cell tumors, or undiagnosed MM may manifest with clinical features that mimic connective tissue diseases. Taking our current results and the above studies into account, the credibility of the antigenic stimulation hypothesis is called into question.

Only one cohort study [39] investigated the effect of the duration of follow-up on the risk of MM and determined that the borderline excess of MM was confined to the early 4-year follow-up. Thomas [10] proposed that although increased risk of MM persisted for hematopoietic cancers throughout the follow-up period, the greatest excess was within the first 3 months after hospitalization. These findings could possibly indicate a common etiologic factor for RA and MM, or further prove the association confirmed in above two studies might be resulted from detection bias. With the current common application of anti-TNF drugs, concern has been raised about the risk of developing malignancy related with their use [52]. However, as implied from two studies mentioned earlier [41], [44], there was no significant increase in the risk of MM among anti-TNF users. Considering the small number of reports available, further studies are warranted to account for this potential confounder when examining the relationship of autoimmune diseases and MM.

Meta-analysis results revealed that pernicious anemia is another risk factor for MM, consistent with several prior epidemiologic studies [53], [54]. The related pathogenesis may involve immune alterations or chromosome abnormalities in the bone marrow of patients with pernicious anemia [54]. On the other hand, vitamin B12 deficiency caused by pernicious anemia may promote MM development by disrupting normal homeostasis for one-carbon metabolism [55]. The lack of association of MM with psoriasis, SLE, SS, and polymyositis/dermatomyositis raises the question of whether oncogenesis is dependent on the type of autoimmune disease. A number of studies evaluating the risk of MM in AS [45] and polymyalgia rheumatic [37] patients have reported positive associations, similar to our assessment of AS and MM. Assumptions such as whether an autoimmune disease resulting in MM is associated with disease activity, amount of autoantibody, and the extent of organ involvement should be examined. Although the current meta-analysis presents a more precise estimate of MM risk in RA patients than individual studies, significant heterogeneity remains a primary limitation of our study. Subgroup analyses failed to determine the source of heterogeneity. The diversity of subject sources and study methodologies may have led to inconsistencies. One such example is the study by Boffetta [33] including 282 patients that died from MM, meaning that they were likely to suffer from more severe diseases and past medical history obtained from their family members was possibly incomplete. Similarly, another study [41] was restricted to subjects older than 65 years whose disease spectrum may be distinct from that of the general population. The variable proportions of races in different studies may additionally contribute to significant heterogeneity. Moreover, misclassification of patients may have occurred in case-control studies, since no consistent diagnosis criteria were applied by investigators and most enrollments were dependent on reports and certificates, rather than specific laboratory data, similar to enrollment in cohort studies. As indicated in cohort studies, RA patients may develop MM after more than 20 years, in which case recall bias may exist in case-control studies.

Another limitation of this study is that certain articles had to be excluded, since no data on MM and RA were available, although their relationship was evaluated. Exclusion of such studies may have led to results bias. In spite of this, the possible bias might not be so evident, since why these data were not reported mostly lied in that authors found no specific association between RA and MM. Although publication bias was borderline significant as indicated by Begg’s test, adjustment for possibly missing manuscripts yielded similar results, strongly supporting the robustness of our analyses.

### Conclusion

Despite its limitations, our meta-analysis provides novel evidence for increased MM development risk in AS and pernicious anemia, although the underlying pathogenesis remains controversial. Notably, RA does not appear to alter the risk of MM, while between-study heterogeneity suggested caution in the interpretation of results. Further large-scale longitudinal studies containing details of risk factors and treatment information are necessary to determine the pathophysiological mechanism linking autoimmune diseases and MM. Moreover, identification of reliable exposure biomarkers of immune stimulation would be significantly beneficial.

## Supporting Information

Appendix S1 **Search strategy for PubMed.**
(DOCX)

Checklist S1 **PRISMA 2009 Checklist for current meta-analysis.**
(DOC)

## References

[1] Kyle RA, Therneau TM, Rajkumar SV, Larson DR, Plevak MF, et al (2004) Incidence of multiple myeloma in Olmsted County, Minnesota - Trend over 6 decades. Cancer 101: 2667–2674. 15481060 10.1002/cncr.20652

[2] Cuzick J (1981) Radiation-Induced Myelomatosis. New England Journal of Medicine 304: 204–210. 7442744 10.1056/NEJM198101223040404

[3] Infante PF (2006) Benzene exposure and multiple myeloma - A detailed meta-analysis of benzene cohort studies. Living In a Chemical World: Framing the Future In Light Of the Past 1076: 90–109. 10.1196/annals.1371.081 17119195

[4] Blair A, Zahm SH, Pearce NE, Heineman EF, Fraumeni JF (1992) Clues to Cancer Etiology from Studies of Farmers. Scandinavian Journal of Work Environment & Health 18: 209–215. 10.5271/sjweh.1578 1411362

[5] Lea AJ (1964) An association between the rheumatic diseases and the reticuloses. Ann Rheum Dis: 480–484. 10.1136/ard.23.6.480 PMC1030963 14229581

[6] Potter M (1986) Plasmacytomas in Mice. Seminars in Oncology 13: 275–281. 3764439

[7] Radl J, Croese JW, Zurcher C, Van den Enden-Vieveen MH, de Leeuw AM (1988) Animal model of human disease. Multiple myeloma. Am J Pathol 132: 593–597. 3414786 PMC1880745

[8] Smedby KE, Vajdic CM, Falster M, Engels EA, Martinez-Maza O, et al (2008) Autoimmune disorders and risk of non-Hodgkin lymphoma subtypes: a pooled analysis within the InterLymph consortium. Blood 111: 4029–4038. 18263783 10.1182/blood-2007-10-119974 PMC2288717

[9] Mellemkjaer L, Pfeiffer RM, Engels EA, Gridley G, Wheeler W, et al (2008) Autoimmune disease in individuals and close family members and susceptibility to non-Hodgkin’s lymphoma. Arthritis and Rheumatism 58: 657–666. 18311836 10.1002/art.23267

[10] Thomas E, Brewster DH, Black RJ, Macfarlane GJ (2000) Risk of malignancy among patients with rheumatic conditions. International Journal of Cancer 88: 497–502. 11054684

[11] Landgren O, Engels EA, Pfeiffer RM, Gridley G, Mellemkjaer L, et al (2006) Autoimmunity and susceptibility to Hodgkin lymphoma: A population-based case-control study in Scandinavia. Journal of the National Cancer Institute 98: 1321–1330. 16985251 10.1093/jnci/djj361

[12] Gridley G, McLaughlin JK, Ekbom A, Klareskog L, Adami HO, et al (1993) Incidence of Cancer among Patients with Rheumatoid-Arthritis. Journal of the National Cancer Institute 85: 307–311. 8426374 10.1093/jnci/85.4.307

[13] Wells G, Shea B, O’Connell D, Peterson J, Welch V, et al. (2011) The Newcastle-Ottawa Scale (NOS) for assessing the quality of nonrandomized studies in meta analyses. Ottawa, ON, Canada: Ottawa Hospital Research Institute. Available: http://www.ohri.ca/programs/clinical_epidemiology/oxford.asp. Accessed 28 December 2013.

[14] Beranger R, Le Cornet C, Schuz J, Fervers B (2013) Occupational and Environmental Exposures Associated with Testicular Germ Cell Tumours: Systematic Review of Prenatal and Life-Long Exposures. Plos One 8. 10.1371/journal.pone.0077130 PMC3796551 24155923

[15] Greenland S (1987) Quantitative Methods In the Review Of Epidemiologic Literature. Epidemiologic Reviews 9: 1–30. 3678409 10.1093/oxfordjournals.epirev.a036298

[16] Siristatidis C, Sergentanis TN, Kanavidis P, Trivella M, Sotiraki M, et al (2013) Controlled ovarian hyperstimulation for IVF: impact on ovarian, endometrial and cervical cancer-a systematic review and meta-analysis. Human Reproduction Update 19: 105–123. 23255514 10.1093/humupd/dms051

[17] Larsson SC, Mantzoros CS, Wolk A (2007) Diabetes mellitus and risk of breast cancer: A meta-analysis. International Journal Of Cancer 121: 856–862. 17397032 10.1002/ijc.22717

[18] Higgins JPT, Thompson SG, Deeks JJ, Altman DG (2003) Measuring inconsistency in meta-analyses. British Medical Journal 327: 557–560. 12958120 10.1136/bmj.327.7414.557 PMC192859

[19] Doody MM, Linet MS, Glass AG, Friedman GD, Pottern LM, et al (1992) Leukemia, lymphoma, and multiple myeloma following selected medical conditions. Cancer Causes Control 3: 449–456. 1525326 10.1007/BF00051358

[20] Bourguet CC, Logue EE (1993) Antigenic stimulation and multiple myeloma. A prospective study. Cancer 72: 2148–2154. 8374872 10.1002/1097-0142(19931001)72:7<2148::aid-cncr2820720714>3.0.co;2-q

[21] Prior P, Symmons DPM, Hawkins CF, Scott DL, Brown R (1984) Cancer Morbidity in Rheumatoid-Arthritis. Annals of the Rheumatic Diseases 43: 128–131. 6712287 10.1136/ard.43.2.128 PMC1001446

[22] Buchbinder R, Barber M, Heuzenroeder L, Wluka AE, Giles G, et al (2008) Incidence of melanoma and other malignancies among rheumatoid arthritis patients treated with methotrexate. Arthritis & Rheumatism-Arthritis Care & Research 59: 794–799. 18512713 10.1002/art.23716

[23] Chen YJ, Chang YT, Wang CB, Wu CY (2011) The Risk of Cancer in Patients With Rheumatoid Arthritis A Nationwide Cohort Study in Taiwan. Arthritis and Rheumatism 63: 352–358. 21279991 10.1002/art.30134

[24] Anderson LA, Gadalla S, Morton LM, Landgren O, Pfeiffer R, et al (2009) Population-based study of autoimmune conditions and the risk of specific lymphoid malignancies. International Journal of Cancer 125: 398–405. 19365835 10.1002/ijc.24287 PMC2692814

[25] Pearce NE, Smith AH, Howard JK, Sheppard RA, Giles HJ, et al (1986) Case-Control Study of Multiple-Myeloma and Farming. British Journal of Cancer 54: 493–500. 3756085 10.1038/bjc.1986.202 PMC2001629

[26] Eriksson M (1993) Rheumatoid arthritis as a risk factor for multiple myeloma: a case-control study. Eur J Cancer 29A: 259–263. 8422292 10.1016/0959-8049(93)90188-l

[27] Landgren O, Linet MS, McMaster ML, Gridley G, Hemminki K, et al (2006) Familial characteristics of autoimmune and hematologic disorders in 8,406 multiple myeloma patients: A population-based case-control study. International Journal of Cancer 118: 3095–3098. 16395700 10.1002/ijc.21745

[28] Askling J, Fored CM, Baecklund E, Brandt L, Backlin C, et al (2005) Haematopoietic malignancies in rheumatoid arthritis: lymphoma risk and characteristics after exposure to tumour necrosis factor antagonists. Annals of the Rheumatic Diseases 64: 1414–1420. 15843454 10.1136/ard.2004.033241 PMC1755232

[29] Hemminki K, Li X, Sundquist K, Sundquist J (2008) Cancer risk in hospitalized rheumatoid arthritis patients. Rheumatology 47: 698–701. 18378514 10.1093/rheumatology/ken130

[30] Isomaki HA, Hakulinen T, Joutsenlahti U (1978) Excess risk of lymphomas, leukemia and myeloma in patients with rheumatoid arthritis. J Chronic Dis 31: 691–696. 730824 10.1016/0021-9681(78)90071-1

[31] Hakulinen T, Isomaki H, Knekt P (1985) Rheumatoid-Arthritis and Cancer Studies Based on Linking Nationwide Registries in Finland. American Journal of Medicine 78: 29–32. 10.1016/0002-9343(85)90242-6 3970037

[32] Cohen HJ, Bernstein RJ, Grufferman S (1987) Role of Immune Stimulation in the Etiology of Multiple-Myeloma - a Case Control Study. American Journal of Hematology 24: 119–126. 3812465 10.1002/ajh.2830240202

[33] Boffetta P, Stellman SD, Garfinkel L (1989) A Case-Control Study of Multiple-Myeloma Nested in the American-Cancer-Society Prospective-Study. International Journal of Cancer 43: 554–559. 2703267 10.1002/ijc.2910430404

[34] Lewis DR, Pottern LM, Brown LM, Silverman DT, Hayes RB, et al (1994) Multiple-Myeloma among Blacks and Whites in the United-States - the Role of Chronic Antigenic-Stimulation. Cancer Causes & Control 5: 529–539. 7827240 10.1007/BF01831381

[35] Vlajinac HD, Pekmezovic TD, Adanja BJ, Marinkovic JM, Kanazir MS, et al (2003) Case-control study of multiple myeloma with special reference to diet as risk factor. Neoplasma 50: 79–83. 12687283

[36] Landgren O, Zhang YW, Zahm SH, Inskip P, Zheng TZ, et al (2006) Risk of multiple myeloma following medication use and medical conditions: A case-control study in Connecticut women. Cancer Epidemiology Biomarkers & Prevention 15: 2342–2347. 10.1158/1055-9965.EPI-06-0097 17132770

[37] Lindqvist EK, Goldin LR, Landgren O, Blimark C, Mellqvist UH, et al (2011) Personal and family history of immune-related conditions increase the risk of plasma cell disorders: a population-based study. Blood 118: 6284–6291. 21998210 10.1182/blood-2011-04-347559 PMC3236117

[38] Isomaki HA, Hakulinen T, Joutsenlahti U (1982) Excess risk of lymphomas, leukemia and myeloma in patients with rheumatoid arthritis. Annals of the Rheumatic Diseases 41: 34–36. 10.1016/0021-9681(78)90071-1 730824

[39] Mellemkjaer L, Linet MS, Gridley G, Frisch M, Moller H, et al (1996) Rheumatoid arthritis and cancer risk. Eur J Cancer 32A: 1753–1757. 8983286 10.1016/0959-8049(96)00210-9

[40] Kauppi M, Pukkala E, Isomaki H (1997) Elevated incidence of hematologic malignancies in patients with Sjogren’s syndrome compared with patients with rheumatoid arthritis (Finland). Cancer Causes Control 8: 201–204. 9134244 10.1023/a:1018472213872

[41] Setoguchi S, Solomon DH, Weinblatt ME, Katz JN, Avorn J, et al (2006) Tumor necrosis factor alpha antagonist use and cancer in patients with rheumatoid arthritis. Arthritis Rheum 54: 2757–2764. 16947774 10.1002/art.22056

[42] Herrinton LJ, Liu L, Shoor S, Mines D (2008) Risk of lymphoproliferative cancer among patients with severe rheumatoid arthritis, 1996–2002. Ann Rheum Dis 67: 574–575. 18349167 10.1136/ard.2007.075986

[43] Parikh-Patel A, White RH, Allen M, Cress R (2009) Risk of cancer among rheumatoid arthritis patients in California. Cancer Causes Control 20: 1001–1010. 19184473 10.1007/s10552-009-9298-y PMC4354853

[44] Dreyer L, Mellemkjaer L, Andersen AR, Bennett P, Poulsen UE, et al (2013) Incidences of overall and site specific cancers in TNF alpha inhibitor treated patients with rheumatoid arthritis and other arthritides - a follow-up study from the DANBIO Registry. Annals of the Rheumatic Diseases 72: 79–82. 22945500 10.1136/annrheumdis-2012-201969

[45] Brown LM, Gridley G, Check D, Landgren O (2008) Risk of multiple myeloma and monoclonal gammopathy of undetermined significance among white and black male United States veterans with prior autoimmune, infectious, inflammatory, and allergic disorders. Blood 111: 3388–3394. 18239085 10.1182/blood-2007-10-121285 PMC2275008

[46] Hemminki K, Liu XD, Forsti A, Ji JG, Sundquist J, et al. (2012) Effect of autoimmune diseases on incidence and survival in subsequent multiple myeloma. Journal of Hematology & Oncology 5. 10.1186/1756-8722-5-59 PMC3473243 23031386

[47] Gramenzi A, Buttino I, Davanzo B, Negri E, Franceschi S, et al (1991) Medical History and the Risk of Multiple-Myeloma. British Journal of Cancer 63: 769–772. 2039702 10.1038/bjc.1991.172 PMC1972384

[48] Cuzick J, Destavola BL (1989) Autoimmune Disorders and Multiple-Myeloma. International Journal of Epidemiology 18: 283–283. 2722380 10.1093/ije/18.1.283

[49] Symmons DPM (1988) Neoplasia in Rheumatoid-Arthritis. Journal of Rheumatology 15: 1319–1322. 3058967

[50] Holly EA, Bracci PM (2003) Population-based study of non-Hodgkin lymphoma, histology, and medical history among human immunodeficiency virus-negative participants in San Francisco. American Journal of Epidemiology 158: 316–327. 12915497 10.1093/aje/kwg145

[51] Bachman TR, Sawitzke AD, Perkins SL, Ward JH, Cannon GW (1996) Methotrexate-associated lymphoma in patients with rheumatoid arthritis - Report of two cases. Arthritis and Rheumatism 39: 325–329. 8849387 10.1002/art.1780390223

[52] Bongartz T, Sutton AJ, Sweeting MJ, Buchan I, Matteson EL, et al (2006) Anti-TNF antibody therapy in rheumatoid arthritis and the risk of serious infections and malignancies: Systematic review and meta-analysis of rare harmful effects in randomized controlled trials (vol 295, pg 2275, 2006). Jama-Journal Of the American Medical Association 295: 2482–2482. 10.1001/jama.295.19.2275 16705109

[53] Hsing AW, Hansson LE, Mclaughlin JK, Nyren O, Blot WJ, et al (1993) Pernicious-Anemia and Subsequent Cancer - a Population-Based Cohort Study. Cancer 71: 745–750. 8431855 10.1002/1097-0142(19930201)71:3<745::aid-cncr2820710316>3.0.co;2-1

[54] Brinton LA, Gridley G, Hrubec Z, Hoover R, Fraumeni JF (1989) Cancer Risk Following Pernicious-Anemia. British Journal of Cancer 59: 810–813. 2736218 10.1038/bjc.1989.169 PMC2247229

[55] Friso S, Choi SW (2005) The potential cocarcinogenic effect of vitamin B12 deficiency. Clin Chem Lab Med 43: 1158–1163. 16197314 10.1515/CCLM.2005.201

