# Supplementary material for: Risk of Multiple Myeloma in Rheumatoid Arthritis: A Meta-Analysis of Case-Control and Cohort Studies
Source: PLoS One. 2014 Mar 13;9(3):e91461. doi: 10.1371/journal.pone.0091461 (PMC3953405; doi:10.1371/journal.pone.0091461)
Supplement: Appendix S1 — Search strategy for PubMed. (DOCX) [file pone.0091461.s001.docx]

Search strategy for PubMed

001 rheumatoid arthritis [MESH Terms]

002 autoimmune diseases [MESH Terms]

003 etiology [MESH Terms]

004 epidemiology [MESH Terms]

005 risk factors [MESH Terms]

006 multiple myeloma [MESH Terms]

007 1 OR 2 OR 3 OR 4 OR 5

008 6 AND 7

009 cancer*[Title]

010 malignanc*[Title]

011 myeloma [Title]

012 9 OR 10 OR 11

013 1 AND 12

014 8 or 13
